# A case series of bread clip ingestions

**DOI:** 10.1093/jscr/rjac163

**Published:** 2022-06-16

**Authors:** Fred J Chuang, Philip J Townend, Michelle L Cooper

**Affiliations:** Department of General Surgery, Gold Coast Health, Gold Coast University Hospital, QLD, Australia; Department of General Surgery, Gold Coast Health, Gold Coast University Hospital, QLD, Australia; Department of General Surgery, Gold Coast Health, Gold Coast University Hospital, QLD, Australia

## Abstract

The bread clip is one of the most insidious foreign body ingested. The bread clip poses a serious medical danger to patients yet may often fail to manifest itself clinically on initial ingestion. We present a case series of three patients with bread clips ingestions that were managed in the Gold Coast University Hospital, Queensland, Australia between 2020 and 2021. Bread clips are not always readily identifiable depending on imaging and the management of these patients will often require a multidisciplinary approach between the surgeons, gastroenterologists and radiologists.

## INTRODUCTION

The bread clip (BC) was originally designed in the early 1950s by Floyd G. Paxton and is a ubiquitous household device used to secure plastic bags. This seemingly innocuous device poses serious medical dangers when inadvertently ingested. BC ingestions may not be obvious on initial presentation and investigation and has a propensity to remain indolent for quite some time before presentation. As a result, the incidence of BC ingestions is unknown.

## METHODS

We present a case series of three patients with BC ingestions. All three patients presented to a tertiary hospital on the Gold Coast, QLD, Australia within a 1-year time frame. To date this is the highest incidence of BC ingestion of any published work.

## RESULTS

All patients were suspected of foreign body (FB) ingestions on initial computed tomography (CT) imaging. The culprit BC was not readily evident until retrieval in two of the tree cases. The results are listed in Table 1. All three patients were from the elderly demographic, where all three had upper dentures. The treatment options, frequency and modality of serial imaging were individualized to each patient. Two were managed surgically and one was managed endoscopically. All patients recovered without complications.

**Table 1:** Patient case summary

	Case 1	Case 2	Case 3
Demographic	72 Female	74 Female	68 Male
Location of BC	Mid-Jejunum	Distal Ileum	D1-Duodenum
Presentation	Incidental	Lower abdominal pain, diarrhoea and vomitting	Epigastric pain, melaena and coffee-ground vomitus
Method	Laparoscopic-assisted small bowel resection and primary anastomosis	Laparoscopic-assisted small bowel resection and primary anastomosis	Upper endoscopic snare retrieval
Dentures	Yes	Yes	Yes
Complications	0	0	0

### Case one

**Figure 1:**
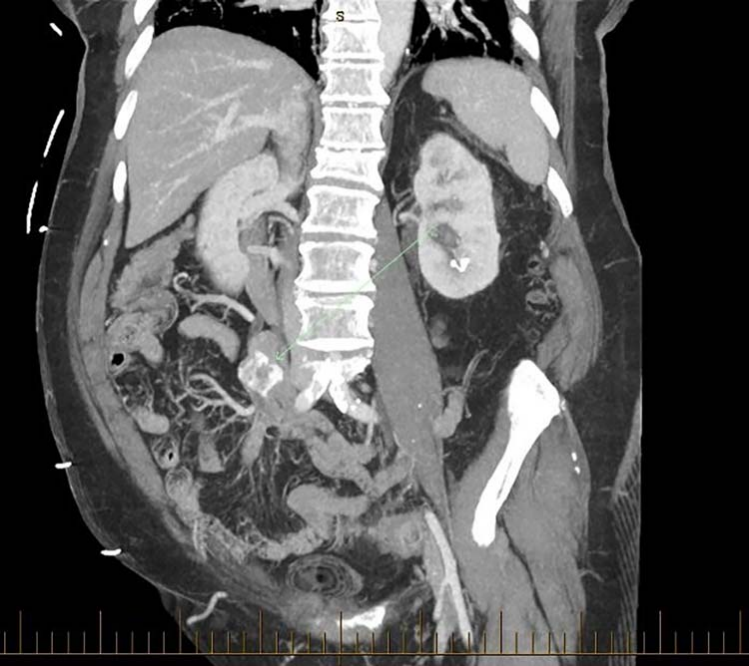
Coronal CT BC embedded in the distal jejunum.

**Figure 2:**
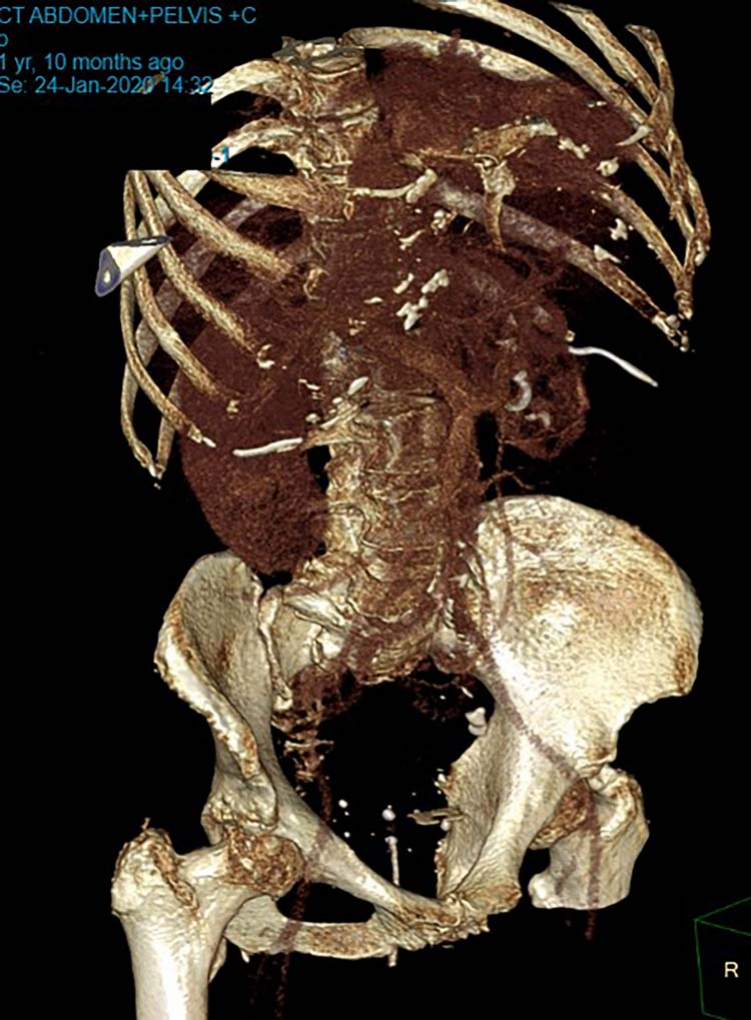
CT reconstructed image, BC seen in lower pelvis.

Patient SC is a 72-year-old lady who presented with urosepsis secondary to a left-sided distal ureteric renal stone. Her medical history includes ischemic heart disease, chronic pulmonary obstructive airway disease, type 2 diabetes mellitus, Parkinson’s disease and a previous open cholecystectomy. She was admitted to the intensive care unit and a left-sided nephrostomy tube was inserted. Initial CT imaging incidentally identified a ‘bread clip’-shaped FB in the distal jejeunum (Figs 1 and 2). There was no evidence of obstruction nor perforation. Abdominal X-rays failed to show the presence of a FB.

The acute surgical unit team consulted the patient and initial conservative management included keeping a stool diary and trialling 1 L of glycoprep. Unfortunately, serial imaging failed to show any meaningful progression of the FB. After a successful antegrade ureteric stent insertion, the patient recovered from urosepsis and a decision was made to proceed with a laparoscopic small bowel resection to retrieve the FB.

Intraoperatively, an inflamed segment of 5 cm of the mid jejunum was identified, the FB was palpable and appeared to be partially eroding through the serosa. A SB resection was performed with primary stapled anastomosis. The FB was identified as a BC.

SC had an uneventful recovery and was discharged from the care of the acute surgical team.

### Case two

**Figure 3:**
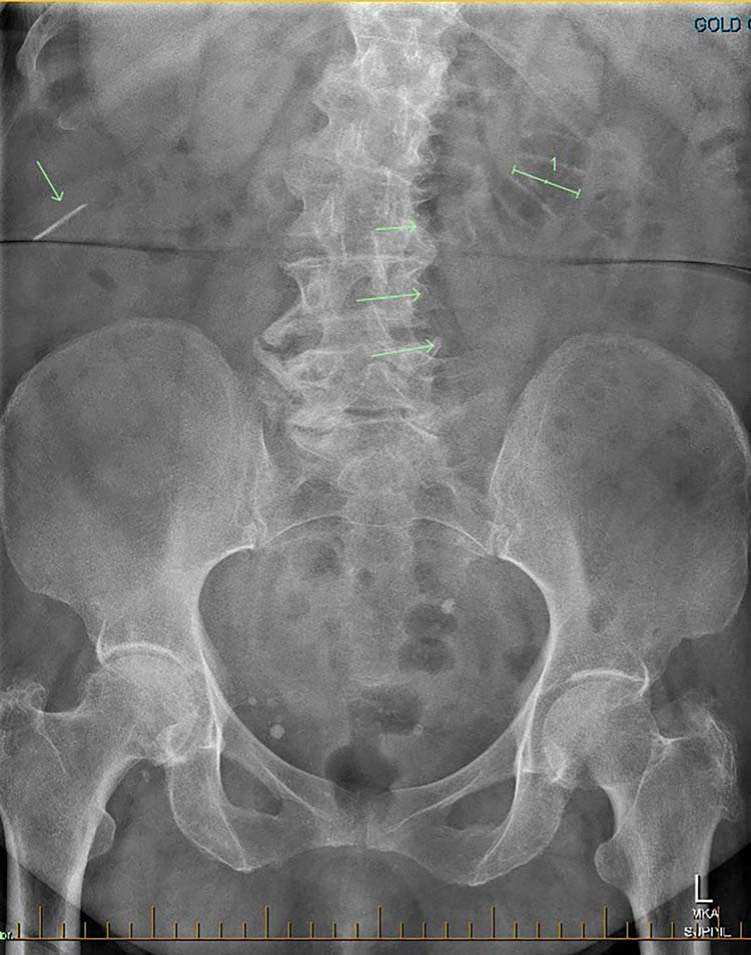
AXR shows a linear hyperdensity in the right upper quadrant.

**Figure 4:**
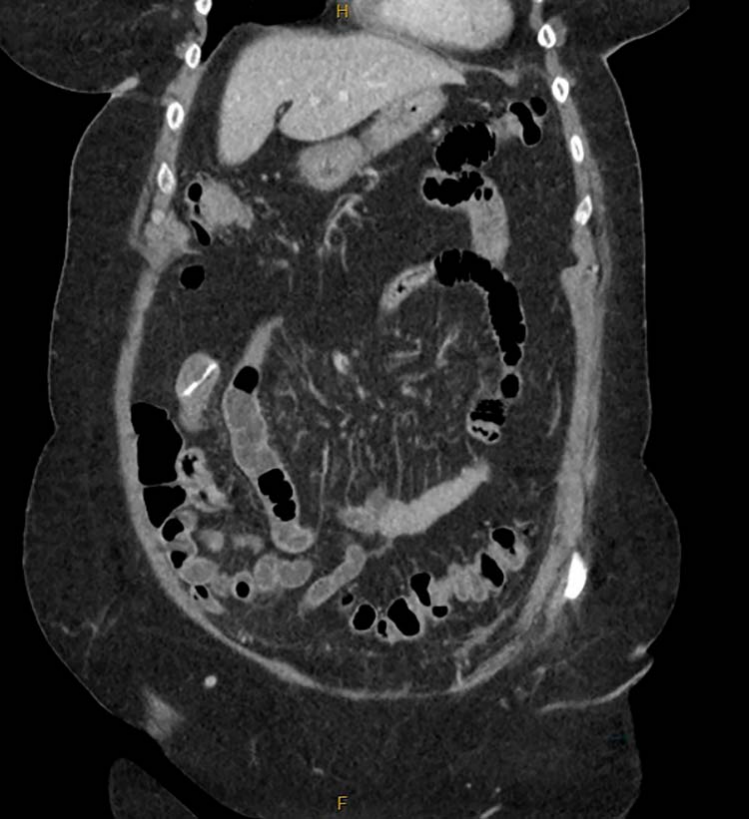
Initial CT imaging for patient RF.

Patient RF a 74-year-old lady with a background of obesity and dyslipidaemia was admitted under the acute surgical care team after experiencing several weeks of diarrhoea, vomiting and a generalized grumbling abdominal pain. Initial abdominal plain films showed a FB in the right middle abdomen (Fig. 3). A follow-up CT was able to highlight this linear hyperdensity which correlated in location to the plain radiographs (Fig. 4). Her laboratory markers were as follows WCC 14 × 10^9^/L, neutrophil count 13 × 10^9^/L, C-reactive protein 57 mg/L. RF herself could not recall a history of FB ingestion. Initially the medical teams had presumed that the FB could have been a fragment of the patients’ denture. RF was placed on a clear fluid diet and daily serial plain films were performed to observe for progression of the FB. On Day 2, interestingly the FB was no longer present on plain films.

Serial CT demonstrated that the FB had progressed, however had embedded itself within a more distal segment of small bowel. A decision was made to resect the affected small bowel and retrieve the FB.

**Figure 5:**
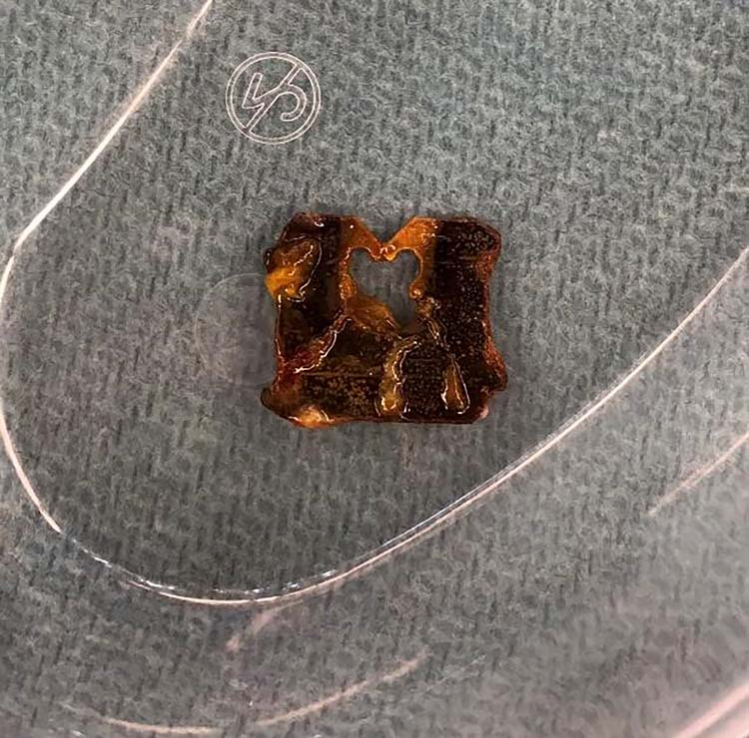
A significantly degraded clip suggested that the object had been present for a prolonged period. An expiration date could not be seen.

A diagnostic laparoscopy was performed and two segments of concerning small bowel were identified. A mini-laparotomy was performed and the FB was palpable at the distal ileum. An enterotomy was performed and the object was identified as a BC (Fig. 5). Due to the inflamed segment and possible impending perforated serosa, the segment of compromised small bowel was resected, and a hand-sewn anastomosis was performed. The small bowel was walked proximally and a further site of compromised bowel suggestive of an impending perforation was identified and the affected site was resected and repaired.

RF was discharged on Day 3 with a 5-day course of oral amoxicillin/clavulanic acid.

### Case 3

Patient RB a 68-year-old gentleman presented to the emergency department with epigastric pain, melaena and coffee ground vomitus. His medical history is significant for ischaemic heart disease, Type 2 diabetes mellitus, Parkinson’s disease and a previous open appendicectomy His initial haemoglobin was 85 g/L, urea was 18 mmol/L. The patient was resuscitated and was given 2 units of packed red blood cells and high dose 80 mg of IV pantoprazole. An urgent upper gastrointestinal endoscopy revealed an entrenched FB at the duodenal bulb measuring 20 mm. A snare and a large biopsy forceps were unable to retrieve the object. Further attempts were abandoned due to the surrounding ulceration and fears of an impending perforation, without being able to identify the FB as it had a metallic appearance.

The acute surgical unit consulted upon the patient and a CT abdomen with oral contrast was performed the next day. CT was unable to accurately identify the position and nature of the foreign object. Filling defects with the stomach fundus and superior part of duodenum were identified, but the FB remained unidentified.

**Figure 6:**
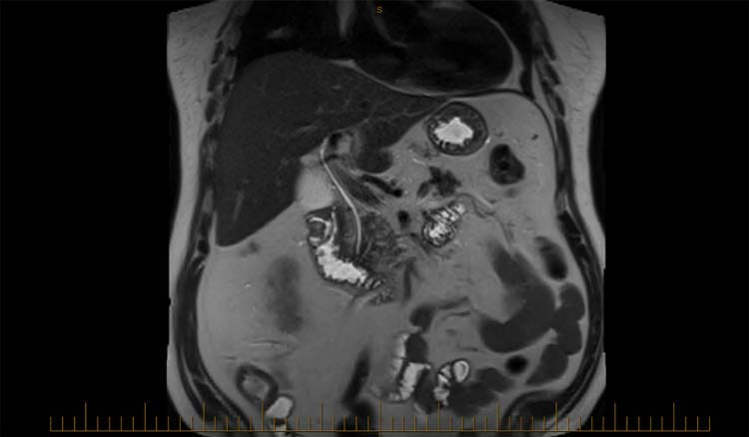
Filling defect within the duodenum on MRCP.

A magnetic cholangiopancreatography (MRCP) was performed to exclude a cholecystoduodenal fistula. A filling defect within the duodenum was identified on the scan; however, there was no evidence of a fistula (Fig. 6).

**Figure 7:**
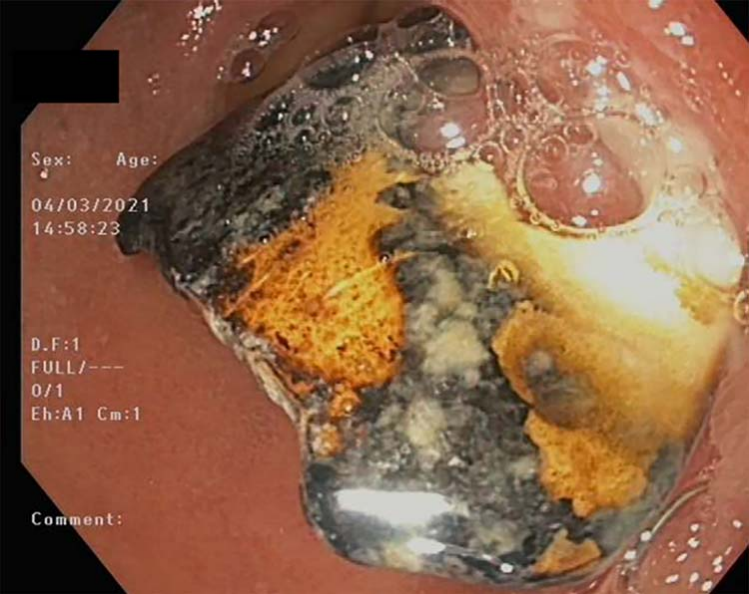
BC embedded in the duodenal mucosa.

On Day 7, a repeat upper gastrointestinal endoscopy was performed. The FB remained in the first part of the duodenum which it had begun to erode through posteriorly (Fig. 7). The endoscopy was fitted with an overtube and with the help of a snare the embedded FB was successfully retrieved.

The patient was successfully discharged, placed on an oral proton pump inhibitors and had an uneventful 8-week follow-up endoscopy.

## DISCUSSION

The literature to date shows that BC ingestions are not uncommon. Decisions for intervention are guided by location of impaction, the type of FB and the clinical presentation of the patient. No published case reports within the last 10 years were able to reporting successful treatment of BC ingestions through conservative means. The morphology of these BCs is unique in that the sharp encircling opening allows the FB to hook and cinch onto mucosa. Prolonged entrapment of mucosa leads to obstruction, ischaemia, bleeding, necrosis and perforation [1–4]. There are many key takeaways from this series.

Firstly, diagnosis itself is often difficult as patients seem to unknowingly consume BCs. Patient’s may remain asymptomatic for long periods of time [3, 5, 6]. Major risk factors include being elderly, edentulous resulting in poor mastication and poor vision [4, 7, 8]. We postulate that dentures decrease the sensation of consumption of foreign bodies leading to cases of unwitting ingestions. Interestingly, in all three cases, the BCs were identified completely intact. All three of our patients were edentulous. Furthermore, two of our patient’s likely had an element of dysphagia secondary to their Parkinson’s disease.

Secondly, as shown in the three cases, unless the BC is calcified, abdominal plain films are not a reliable method of tracking nor diagnosing BC ingestion [9–13]. CT is more sensitive, with a sensitivity rate of 67%, but is not consistently reliable on its own [6, 7]. Reconstructed 3D imaging is effective in identifying BCs. Most BCs are made of low-density polystyrene, a type of versatile solid plastic, which are not readily identified on plain film imaging [14, 15]. This non-degradable plastic means that it is usually only a matter of time before a patient develops a complication.

Thirdly, endoscopic removal can be quite difficult and multiple techniques may be incorporated for a successful retrieval. Methods previously cited including snapping the clip and retrieving it in parts with a gastric band cutter. Alternatively, using biopsy forceps with a view to grasp advance and then disengaging or using a snare to alter the shape of the clip are other creative retrieval methods [1, 16, 17].

Finally, from a public health point of view, BCs although seemingly harmless can cause significant morbidity especially in the elderly population. Although the incidence of BC ingestion is unknown in Australia, a chain of BC presentations to a single tertiary hospital within a short time frame raises the suspicion that the incidence is higher than presumed. Serious discussions regarding changes to food industry packaging practises within Australia should be considered and alternatives such as larger plastics clips or sealing tapes should be recommended.
